# Analysis on Entrepreneurship Psychology of Preschool Education Students With Entrepreneurial Intention

**DOI:** 10.3389/fpsyg.2020.01559

**Published:** 2020-07-09

**Authors:** Yiwei Yin, Liu Yang, Bojing Liu

**Affiliations:** ^1^Party Committee Teacher Work Department, Yibin University, Yibin, China; ^2^Faculty of Music and Performing Arts, Sultan Idris Education University, Tanjung Malim, Malaysia; ^3^School of International Studies, Wenzhou Medical University, Wenzhou, China

**Keywords:** entrepreneurial psychology, preschool education major, entrepreneurial intention, psychological quality, psychological support

## Abstract

To diversify the creative thinking of preschool education students and improve their ability to innovate and start a business, a survey of preschool education students under entrepreneurial psychology theory was conducted in this research. Based on the theoretical foundation of entrepreneurial psychology, this article analyzed the entrepreneurial psychological quality and psychological education of college students. By investigating preschool education students in a certain college in Sichuan as the research object, the author explored the current entrepreneurial intentions of college students and their entrepreneurial psychological problems. In response to the current entrepreneurial situation of college graduates, relevant countermeasures were proposed from the perspective of the school to support their entrepreneurial psychology. Among the 205 preschool education college students, the students were more willing to give full play to their professional expertise in terms of employment intentions. At the same time, there were still situations in which students were dissatisfied with the prospects of preschool education career development and wanted to achieve the value of life through other approaches. Most students in preschool education had a wait-and-see attitude toward entrepreneurship. Only 35% of students had a clear intention to start a business and made their plans for entrepreneurship. More than 90% of students held that they had developed inadequate entrepreneurial ability, and 80% of students believed that they lacked the required professional knowledge. These two factors constitute the main reasons for students’ negative attitudes toward entrepreneurship. Nevertheless, colleges may stimulate the potential of students’ self-development through the improvement of entrepreneurial psychological education courses, the construction of psychological consultation institutions on campus, and the establishment of interactive platforms for entrepreneurship. In this way, students’ entrepreneurial psychology can be cultivated in an all-round way. Therefore, to deal with the weak overall entrepreneurial consciousness of college students, the colleges should cultivate entrepreneurial innovative talents by strengthening the psychological education of entrepreneurship for students, and help college students achieve entrepreneurial success.

## Introduction

The choice of college students to start their own businesses is of great strategic significance to solve the employment issue in China. Chinese Premier Li Keqiang has also reiterated that the core of “mass entrepreneurship and innovation” lies in stimulating the creativity of people, especially in stimulating the creativity of young people (McNally et al., 2016). Employment driven by entrepreneurship can not only fundamentally solve the problem of difficult employment for college students and alleviate the national employment burden but also the vitality of entrepreneurship will create a multiplier effect that drives employment. Entrepreneurial ability is a person’s ability to survive and develop in entrepreneurial practice. Through self-employment, college graduates can closely combine their interests with their careers to do what they are most interested in and are willing to do what they think is the most worthwhile (Yang, 2018). In the current society, college students are encouraged to start their own businesses. From the perspective of college students themselves, the main driving force for their entrepreneurship is to seek the self-fulfillment. Through entrepreneurship and entrepreneurial practice, college students are able to fully mobilize their subjective initiative, change their employment mentality, and learn to regulate and control themselves.

Entrepreneurial psychology is a special psychological phenomenon manifested during the course of entrepreneurial behavior, that is, the mental state of the entrepreneur’s regulation and dominance of entrepreneurial behavior in entrepreneurial activities. In the process of entrepreneurship, college students can give full play to their subjective initiative and creativity, reflect their entrepreneurial ability, and ultimately realize the value of life. From a psychological perspective, ability is one of the important factors of individual psychological phenomena. Individual ability, individual cognition, and individual emotion are interdependent and together form a person’s psychology. In the process of starting a business for college students, there is no doubt that they must face pressure from various parties, such as lack of experience, interpersonal limitations, and shortage of funds. Therefore, college entrepreneurs must be able to maintain stress resistance at all times to face difficulties and unknown risks in the process of entrepreneurship (Ehrlin et al., 2016). Only by maintaining a stable entrepreneurial mood can they guarantee the unwavering willingness to start a business, support individual entrepreneurial goals and implement entrepreneurial behaviors. In the process, they continuously improve the ability to enable entrepreneurial behaviors to be successfully completed.

In the era of vigorous development of college students’ mass entrepreneurship and innovation, preschool education, as the basis of the entire education system, plays an important role in cultivating students’ innovation and entrepreneurship ability in personal development and social progress. In summary, this article analyzes the entrepreneurial psychological quality and entrepreneurial psychological education of college students from the perspective of entrepreneurial psychology. By taking preschool education students in one college in Jiangsu as the research object, this article investigates the current entrepreneurial intentions of college students. The entrepreneurial psychological problems existing in college students are analyzed and the corresponding psychological support suggestions are proposed to disperse the creative thinking of preschool education students and enhance their innovative and entrepreneurial ability.

## Literature Review

Maslow believes that everyone has a tendency to pursue self-realization and engage in creative behavior. Psychologist Williams believes that people with creative tendencies have the four characteristics of curiosity, imagination, challenge, and adventure. Some scholars in China believe that the creative tendency is the creative personality, which means that people have a positive psychological tendency toward creative activities, which is an important part of creativity (Duman, 2018). Some studies have also pointed out that creativity training includes creative cognitive behavior and creative affective behavior. Creative affective behavior refers to the personality traits exhibited by individuals during activities, and this personality trait is a positive psychological tendency. Therefore, creative affective behavior is also called creative tendency (Woodside et al., 2016).

Emotion is a kind of psychological experience, which reflects the relationship between the individual and other things. In this relationship, the individual’s needs are used as an intermediary. If entrepreneurship can meet the needs of college students, a positive emotional experience will be produced, which will promote entrepreneurship. If entrepreneurship does not meet the needs of college students, a disgusted emotional experience will be produced, which undoubtedly has a negative effect on college students’ entrepreneurial behavior (Asghar et al., 2018). Research shows that positive emotions have a positive impact on entrepreneurs’ judgments, decision-making, tendencies, entrepreneurial intentions, enthusiasm, creativity, and successful establishment of enterprises. Also, positive emotions can increase their attention and energy (Wang et al., 2018). For research on college students’ entrepreneurial tendencies, Watchravesringkan et al. (2013) believed that the self-fulfillment of personal values has a significant positive impact on entrepreneurial attitudes or entrepreneurial tendencies. The entrepreneurial knowledge and skills possessed by individuals have a moderating role in their influencing relationship. The higher the individual’s sense of self-efficacy, the higher the probability of entrepreneurship. Moreover, self-actualization, cognition, and role will have a great impact on college students’ entrepreneurial tendencies.

In summary, regarding the entrepreneurial issues of college students, the academic community has explored from the perspectives of entrepreneurial emotions. The research angle and the emphasis are different. According to the existing research, for current entrepreneurial activities of college students, entrepreneurial psychological quality is an important factor in determining entrepreneurial behavior in terms of cognition, emotion, will and tendency (Shen et al., 2019). At present, there are few studies on entrepreneurial intentions of students in a specific major. Therefore, based on entrepreneurial psychology, this article discusses the entrepreneurial intentions of preschool education students in the industry.

## Materials and Methods

### College Students’ Entrepreneurial Psychology and Education

**FIGURE 1:**
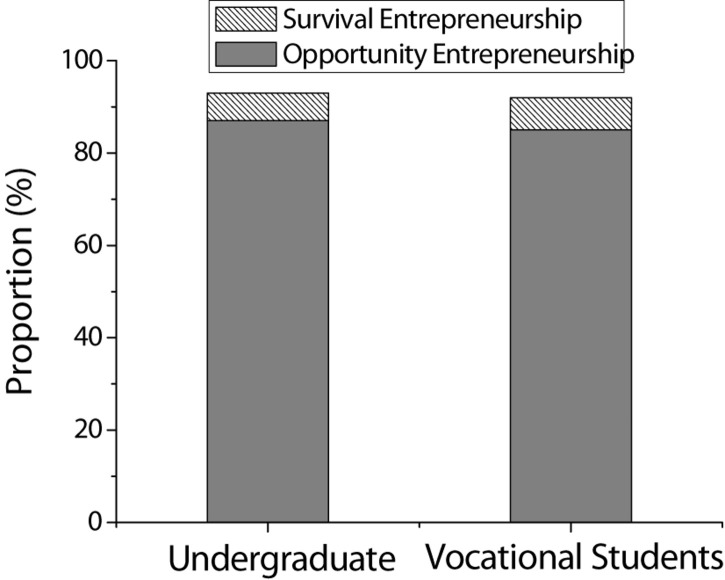
Specific entrepreneurial types of undergraduate and vocational students.

At present, China’s college education is expanding and the number of college graduates is increasing year by year. It is expected that the number of college graduates will reach a record high in 2020, reaching 8.74 million. In contrast, China’s economic and cultural development has entered a new normal, and the rapid development of the market economy has made society’s demand for labor less and less. It is followed by great employment pressure in the whole society. It is also the reason why many college students embark on the path of self-employment. In the past 5 years, the proportion of Chinese college students’ entrepreneurship has steadily increased. Nearly 40% of college graduates who choose to start a business have a rural family background. Education and retail sectors are their main fields of entrepreneurship. The proportion of self-employment in undergraduates and vocational students increased from 1.2 and 2.9% in 2012 to 2.1 and 3.9% in 2016. The vast majority of graduates who choose to start their businesses are “opportunistic entrepreneurship,” that is, to start a business to seize and use market opportunities. A few belong to “survival entrepreneurship,” that is, to start a business due to failure in finding suitable jobs. The specific situation of undergraduates and vocational students choosing self-employment is shown in Figure 1.

As entrepreneurs, they usually have the characteristics of divergent thinking; they are dissatisfied with the *status quo* and are not superstitious about authority. Thus, they generally have innovative psychology. Human psychology mainly includes consciousness, will, ability, and personality. From the perspective of psychology, entrepreneurial psychology is in a special state, which refers to a psychological phenomenon manifested by entrepreneurs in entrepreneurial behavior. The entrepreneurial psychology will regulate and control entrepreneurial behavior. Entrepreneurship is a process full of unknowns and challenges. Especially for college students, they are relatively inadequate in terms of capital reserves, market control, and experience accumulation. Therefore, it is important to maintain a stable psychological mood. In the background of “mass entrepreneurship and innovation,” the difficult entrepreneurial process has higher requirements for the psychological qualities of entrepreneurs. College students must have entrepreneurial professional abilities and stress resistance to deal with entrepreneurial setbacks and difficulties, thereby improving mental capacity to accept unknown challenges. From the perspective of psychological constituents, college students need to constantly update the entrepreneurial cognition, maintain a stable entrepreneurial mood, and ensure that the entrepreneurial will is unshakable during the entrepreneurial process. In this way, they can continuously improve their ability and make entrepreneurial behavior successful.

A special psychological state reflected in entrepreneurial behavior is that entrepreneurial cognition is the basis for individuals to start entrepreneurial behaviors. At the same time, entrepreneurial behavior and language are jointly regulated by various psychological factors such as emotions, will, ability and personality. Entrepreneurial emotion can promote the development of entrepreneurial behavior. The entrepreneurial will, as the cornerstone of the entire entrepreneurial behavior, ultimately promotes the achievement of goals (Asoni and Sanandaji, 2016; Li, 2017; Pallesen, 2018). For college entrepreneurs, the psychological quality of entrepreneurship is not only a general characteristic of psychological conditions in entrepreneurial behavior but also a comprehensive psychological quality formed by entrepreneurial individuals under the influence of external environmental factors. Therefore, it is necessary for colleges to combine psychology-related theories to carry out entrepreneurship psychological education for college students and cultivate good psychological qualities of college entrepreneurs. Entrepreneurship psychological education in colleges should be based on the principle of fostering entrepreneurial personality of college students. Teachers should inspire students’ entrepreneurial thinking and cultivate students’ entrepreneurial interests through guidance. At the same time, they need to exercise students’ entrepreneurial will, so that they can respond to the success and failure in the entrepreneurial process with a good and stable entrepreneurial emotion (Wu et al., 2018). In addition, colleges should combine with the school-running characteristics, strengthen the entrepreneurial psychological education in addition to the professional education, and help students identify entrepreneurial goals; explore entrepreneurial potential, cultivate the entrepreneurial spirit, and shape good entrepreneurial psychological qualities.

Entrepreneurial psychological education for college students is the key to implementing entrepreneurship education. The entrepreneurial psychological education of Chinese colleges is still in the initial stage of development. In combination with China’s economic and political development line, in the process of psychological education for college entrepreneurs, it is necessary to grasp “Chinese characteristics” and draw lessons from overseas relatively mature entrepreneurial psychological education for college students. Then, a mode suitable for college students’ entrepreneurial psychological education can be explored in China.

### College Students’ Entrepreneurial Psychological Quality

College students’ entrepreneurial psychological quality is one of the necessary conditions for entrepreneurs, and it plays an important role in ensuring the success of college students’ entrepreneurship. Entrepreneur’s success is the result of a combination of factors. Psychological quality has an important impact on entrepreneurial success, and it is a firm belief that supports entrepreneurs’ aggressiveness and success (Li et al., 2019). In the human psychological process, the creative tendency is an indispensable psychological quality of creative talents. It plays an important role in entrepreneurial behavior and guides creativity through promotion, regulation and other methods. It is precisely because of the creative tendency that individual creativity develops in a clear direction. The personality characteristic of entrepreneurs is also an important quality in entrepreneurial psychological qualities, which can reflect the entrepreneurial spirit and affect the entire process of entrepreneurs’ implementation of specific entrepreneurial behaviors.

The personality characteristics of entrepreneurs are the sum of the essential and stable psychological characteristics embodied by the entrepreneurs. It is the embodiment of the entrepreneur’s mental outlook. The personality characteristics of entrepreneurs deeply affect the whole process of entrepreneurs’ specific entrepreneurial behaviors, and they are the essential psychological qualities of entrepreneurs to achieve successful entrepreneurship. First, self-confidence is the basic factor that affects the success of entrepreneurs and is also a prerequisite for the formation and development of other personality characteristics. It plays a decisive role in many other aspects of good psychological quality. Second, the independent spirit is the first psychological quality that entrepreneurs should possess. Entrepreneurs can make their judgments decisively without being influenced by the complex external environment. At the same time, they should be good at cooperating with others to realize the optimization and integration of resources, thereby creating a win-win harmony and achieving entrepreneurial success. Third, firm will and keen judgment are the psychological qualities that entrepreneurs must possess. Entrepreneurs must be able to stick to their beliefs, find the causes of problems and find solutions to them, thereby turning crises into opportunities. Fourth is the spirit of adventure, innovation, and responsibility.

Entrepreneurial motivation is the idea of conducting behavioral activities to meet the needs of the realization of personal value, and it is also the internal reason and motivation to encourage entrepreneurs to achieve their goals (Zhang and Wei, 2017). For college entrepreneurs, accordingly, aggressive entrepreneurial motivation is the basic psychological measure to implement specific measures. Generally, college students can gain a career in society through the accumulation of knowledge and skills after studying at college, and reasonable personal positioning. Therefore, few college students have firm entrepreneurial motivations and beliefs. Usually, an entrepreneur is lonely. When facing the setbacks in entrepreneurship, the entrepreneur must have hard-working determination and perseverance. This unremitting entrepreneurial emotion may promote people to think independently and solve problems with a scientific thinking system. In the actual entrepreneurial process, enterprises will face various difficulties and unknown risks during the rising period. Optimistic entrepreneurial confidence is a necessary psychological quality (Park and Kim, 2017; Virginia and Carlos, 2018). Although the entrepreneurs have a difficult period initially, they must maintain a stable mentality and full self-confidence at this time. When transitioning to a stable period of development, entrepreneurs should also be free from arrogance and impetuosity, persist in innovation, and grasp the direction of progress. If an enterprise enters a period of decline in development, it is even more necessary to have the conviction of being more frustrated and more courageous, as well as sum up experience and lessons. Maintaining the necessary confidence in oneself is an indispensable attitude.

### Research Design

Research Object: In this study, the preschool education students from a college in Sichuan as the research object were selected to conduct a questionnaire survey. Also, 212 students in the senior grade of the major were selected to issue questionnaires to understand the college students’ entrepreneurial intention and entrepreneurial psychological quality. The questionnaire of this research was based on the theory of entrepreneurship of college students. Under the reference and summary of the existing questionnaires about the employment intention of preschool education graduates, the questionnaire of the *Preschool Education Employment Intention Survey* was compiled. Among them, there were 6 questions in career attitude and 2 questions in entrepreneurial intention. The Likert scale was used to measure the survey subjects, and the results were five categorical variables: 1–5 scores indicate “strongly disagree,” “disagree,” “not necessarily,” “agree,” and “strongly agree” on the content of the statement, respectively (Jung and Sung, 2016). Reliability analysis shows that Cronbach’s α value is 0.89, which is >0.6, indicating that the overall reliability level of the scale is high.

A total of 212 questionnaires were distributed in this survey, and 209 were recovered, of which 205 were valid questionnaires, and the effective recovery rate was 96.7%. SPSS 24.0 was used to perform descriptive statistics on the test samples, and the independent sample *t*-test was performed.

## Results

### Career Attitude and Entrepreneurial Intention of Preschool Education Students

**FIGURE 2:**
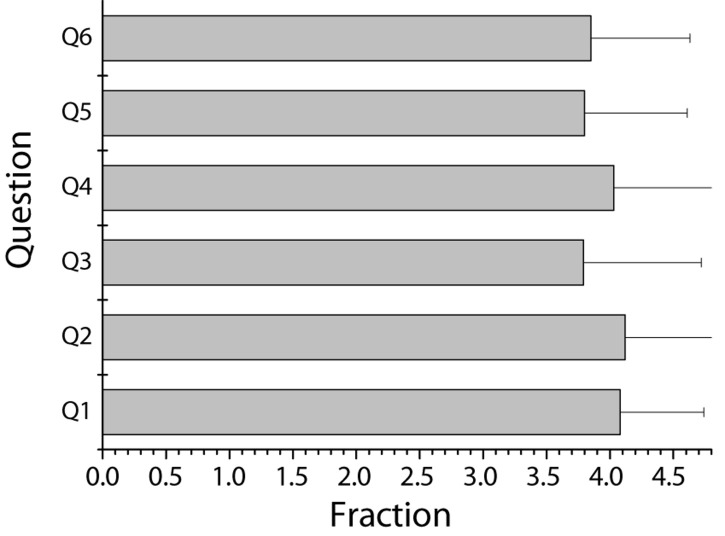
Statistics of students’ career attitudes.

Of the 205 preschool education students surveyed, 7 are males and 198 are females. A total of 73 students were born in cities and 132 in rural areas. Besides, the parents of 17 students are civil servants in public institutions, the parents of 6 students are enterprise workers, 21 are self-employed, 126 are farmers working at home, and 35 are other occupations. Descriptive analysis and statistical results of the career attitudes of preschool education students in this study are shown in Table 1 and Figure 2, respectively, for a total of 6 questions. According to results in the table, students generally gave higher scores in two aspects of “having a great sense of achievement in preschool education” and “having all kinds of skills engage in preschool education-related occupations.” There was a lower level of recognition in the aspect of “engaging in preschool education-related occupations could realize one’s own life value.” Obviously, preschool education students were more willing to give play to their professional expertise and reflect their knowledge and skills. At the same time, however, there were still situations in which students were dissatisfied with the prospects of preschool education career development and wanted to achieve the value of life by other means. Although this group of students was small in number, they were subjects with entrepreneurial potential.

**TABLE 1:** Descriptive analysis of career attitudes (*n* = 205).

Question	Minimum	Maximum	Mean	Standard deviation
Q1: I have a great sense of achievement in preschool education	1	5	4.08	0.66
Q2: I have all kinds of skills to engage in preschool education-related occupations	1	5	4.12	0.74
Q3: Engaging in preschool education-related occupations can realize one’s own life value	1	5	3.79	0.93
Q4: I like to deal with preschool children	1	5	4.03	0.87
Q5: My career choice is related to preschool education	1	5	3.80	0.81
Q6: I like the job related to preschool education	1	5	3.85	0.78

**FIGURE 3:**
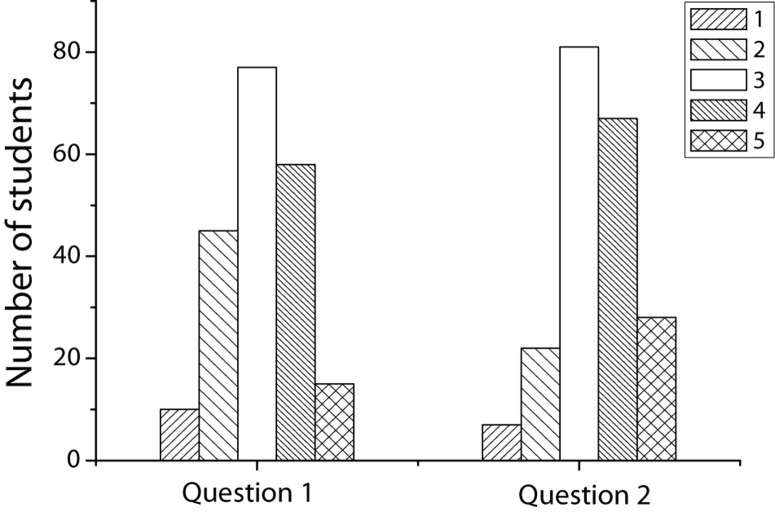
The survey results of entrepreneurial intentions.

About the survey of students’ entrepreneurial intentions, this article involves two questions. The first question is “I will start a business after graduation” and the second question is “If the time is mature after graduation, I will consider starting a business.” The survey results are shown in Figure 3. The average of the first question is 3.11, and the average of the second question is 3.42. It can be seen that for college students in preschool education, most students have a wait-and-see and uncertain attitude about entrepreneurship. Only 35% of students have a clear intention to start a business and have their plans for entrepreneurship.

### Analysis of Entrepreneurial Psychological Problems of College Students

**FIGURE 4:**
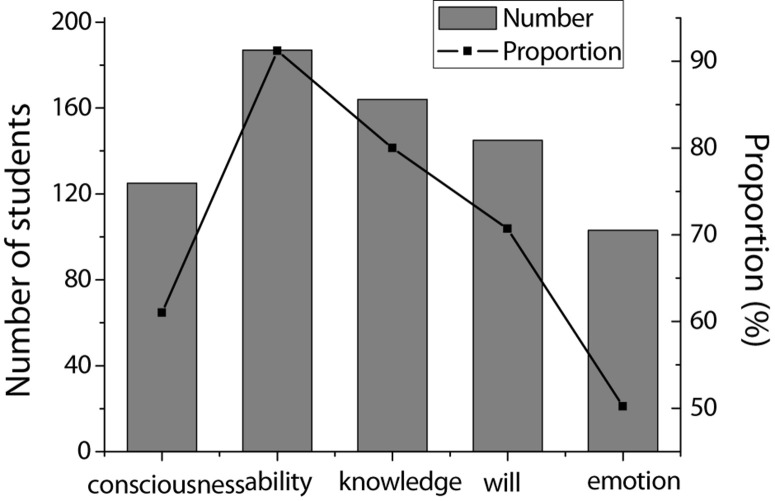
Statistics of the qualities need to be improved for entrepreneurship.

In the questionnaire, a multiple-choice question was designed: If choosing to start a business after graduation, what do you think you need to improve yourself, including five options of entrepreneurial consciousness, entrepreneurial ability, entrepreneurial knowledge, entrepreneurial will, and entrepreneurial emotion. The survey results are shown in Table 2 and Figure 4.

**TABLE 2:** Survey of the qualities need to be improved for entrepreneurship.

Content	Number of students	Proportion (%)
Entrepreneurial consciousness	125	61.0
Entrepreneurial ability	187	91.2
Entrepreneurial knowledge	164	80.0
Entrepreneurial will	145	70.7
Entrepreneurial emotion	103	50.2

The survey results show that more than 90% of students believe that their entrepreneurial ability is weak, which is also the highest proportion of all entrepreneurial qualities that need to be strengthened. It also means that the most significant one of the entrepreneurial psychological problems of college students is that entrepreneurial ability is relatively low. At present, there are fewer successful cases of overall entrepreneurship for college students in China. It has a lot to do with the lack of external abilities. Entrepreneurs are less able to withstand stress and resolve the crisis when they encounter difficulties. Fresh graduates will be more confused and caught off guard, and they are eager to escape and give up, eventually leading to entrepreneurial failure. Then, 80% of students believe that their professional knowledge is lacking, and knowledge can directly promote people’s behavioral decisions. Entrepreneurs must have comprehensive entrepreneurial knowledge, including operations, finance, law, and market economy, to ensure that the entire entrepreneurial behavior is carried out in accordance with the correct plan.

It is found in the analysis of the entrepreneurial psychological problems of college students that the instability of entrepreneurship is also an important factor affecting the entrepreneurial intention. The will of human has the characteristics of consciousness, decisiveness, and tenacity. It is an excellent psychological quality for people to carry out activities, regulate behaviors, and finally realize their ideals. At present, college entrepreneurs in China are generally mentally unstable, usually short-tempered and impatient, easily leading to the failure of entrepreneurship and difficulty in forming an inertial abandonment thinking.

The survey also finds that students have entrepreneurial psychological problems such as weak entrepreneurial consciousness and entrepreneurial emotion. The traditional employment survival consciousness has a profound impact on the entrepreneurial consciousness of college graduates. At present, most college students still have a dependence on the state management institution. Entrepreneurial consciousness, as a new idea in the new era, will promote entrepreneurial behavior. The root cause of entrepreneurial psychological problems among college students is the lack of entrepreneurial consciousness. Due to the lack of entrepreneurial motivation, ideals, and beliefs of modern college students, entrepreneurial emotion is not strong. A large proportion of college entrepreneurs are impulsive entrepreneurs, and they can continue to struggle in favorable circumstances. However, when they encounter setbacks, they mostly choose to escape due to a lack of entrepreneurial emotional support, resulting in failure to achieve personal value and entrepreneurial ideals.

### Relevant Countermeasures for College Students’ Entrepreneurial Psychological Support

**FIGURE 5:**
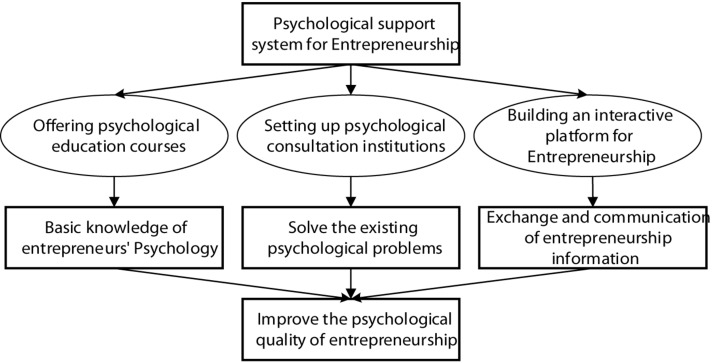
Entrepreneurship psychological support system for college students.

Improving college students’ entrepreneurial enthusiasm and stimulating entrepreneurial consciousness are important directions for colleges to cultivate students’ innovative entrepreneurial thinking. Colleges should take self-construction as the starting point, and propose relevant psychological support countermeasures against their entrepreneurial psychological problems. Entrepreneurship psychological support mainly includes three aspects: offering psychological education courses, setting up psychological consultation institutions, and building an interactive platform for entrepreneurship, as shown in Figure 5.

At present, China’s colleges have offered entrepreneurship-related courses that mainly provide college students with entrepreneurial-related knowledge. However, there is little education related to entrepreneurial psychology for students, and it is thus necessary to provide relevant courses on campus to enable students with entrepreneurial ideas to master the basic common sense related to entrepreneurial psychology, thereby improving the entrepreneurial quality of the students in this part. Then, to address the psychological contradictions generated by college entrepreneurs in the process of entrepreneurship, special counseling can be conducted through the construction of psychological consultation institutions on campus. Psychological consultation institutions are mainly to help college entrepreneurs to relieve stress, find potential psychological problems and solve existing psychological obstacles. For college students, they can offer psychological security. Finally, a more meaningful measure is to build an interactive platform for entrepreneurship to help college students communicate entrepreneurial information through WeChat, Weibo, and other platforms. In this way, the doubts and difficulties can be resolved in the process of entrepreneurship, thereby creating a good entrepreneurial communication atmosphere.

## Discussion

China has entered a new normal of economic and social development. The government has also issued relevant policy support to college students with entrepreneurial intentions and abilities. Entrepreneurship, as a systematic project, involves many aspects such as entrepreneurial consciousness, entrepreneurial ability, and policy support. A series of problems that arise during the entrepreneurial process are also a comprehensive test of the psychological qualities of entrepreneurs (Baloglu, 2017; Wu et al., 2019). In the context of the country’s explicit promotion of entrepreneurship, colleges should provide corresponding help and support to entrepreneurs. However, the entire society has not yet formed the consciousness of psychological education for entrepreneurs, including the cultivation of psychological qualities in the early stages of entrepreneurship, and the firm belief in coping with setbacks in entrepreneurship. The entrepreneurial psychological education environment for college students in China continues to improve, so that entrepreneurial psychological education can serve the purpose of helping college students to increase entrepreneurship rate (Wu and Wu, 2017; Li et al., 2019). Positive personality traits play an important and far-reaching role in entrepreneurial learning and entrepreneurial intentions. It requires college students to have a clear understanding of themselves, consult relevant personnel when encountering entrepreneurial problems, participate in relevant entrepreneurial activities in schools and society as much as possible, and enhance entrepreneurial confidence, gradually forming a good situation for mass entrepreneurship and innovation.

Based on the theoretical basis of entrepreneurial psychology, the entrepreneurial psychological quality and psychological education of college students are analyzed in this study. By investigating preschool education students in one college in Sichuan as the research object, this article explores the current entrepreneurial intentions of college students and their entrepreneurial psychological problems (Zhu et al., 2019). Among the 205 preschool education college students surveyed, in terms of employment intentions, the students were more willing to give play to their professional expertise and reflect their knowledge and skills. At the same time, however, there are still situations in which students were dissatisfied with the prospects of preschool education career development and wanted to realize the value of life by other means. After summing up the students’ intentions for entrepreneurship, the author found that most of them have a wait-and-see and uncertain attitude about entrepreneurship (Chen, 2019). Only 35% of students had a clearer intention to start a business and made their plans for entrepreneurship. Besides, more than 90% of students held that they had developed inadequate entrepreneurial ability, and 80% of students believed that they lacked the required professional knowledge. These two factors were the main reasons for students to have a negative attitude toward entrepreneurship. Therefore, while improving the professional level of students from a college perspective, the college should pay attention to the entrepreneurial psychological problems of students (Obschonka et al., 2018). Under the background of the era of vigorously strengthening ideological and political education in colleges across the country, the entrepreneurial psychological quality of college students is considered from the perspective of entrepreneurship education. It helps to cultivate the innovation and entrepreneurship spirit of college students. Also, it is also important to encourage and guide college students’ entrepreneurial practice activities. The concept of entrepreneurial psychological is derived from the concept of general psychology. According to the cognition, emotion and will of general psychology, the characteristics of college students’ entrepreneurial cognition, entrepreneurial emotion, entrepreneurial will and entrepreneurial personality are analyzed systematically. Thereby, the theoretical basis of entrepreneurial psychological knowledge has a systematic nature similar to the order in which general psychology is produced.

As for colleges, it is necessary to carry out entrepreneurial psychological education for students, and teach students the necessary skills to start a business and how to strengthen their entrepreneurial consciousness (Shen and Ho, 2020; Wu et al., 2020). Colleges can stimulate the potential of students’ self-development through the improvement of entrepreneurial psychological education courses, the construction of psychological consultation institutions on campus, and the establishment of interactive platforms for entrepreneurship. Then, students’ entrepreneurial psychology can be cultivated in an all-round way. This article has made a certain contribution to the promotion of entrepreneurial psychological education for college students. Nevertheless, this research is mainly aimed at the investigation of preschool education students (Zheng et al., 2015; Chen, 2018), leading to limited scope of the survey samples. Therefore, the sample size should be expanded for analysis in future research.

## Conclusion

This article further analyzes and promotes the theoretical research and practical value of entrepreneurial psychological education for college students. It constructs a complete methods and strategies for the internal knowledge system and external environment intervention of college students’ entrepreneurial psychological education. It provides a theoretical basis for how to further develop college students’ entrepreneurial psychological education in China’s higher education. This article comprehensively analyzed the concepts of entrepreneurial learning, personality traits and entrepreneurial intentions, as well as studies related theories. Also, the general characteristics and influencing factors of employment intention of preschool education graduates were discussed. The theory of planned behavior was used to analyze the reasons for the employment intention of pre-school education graduates (Liu and Chen, 2018). Self-employment is a new trend for college students to choose a career, and it is also a development demand for the country’s prosperity and social progress. College students as entrepreneurial potential stocks should have good entrepreneurial psychology. However, the overall entrepreneurial consciousness of college students is still relatively weak, and their understanding of entrepreneurial psychology is not comprehensive. Therefore, the colleges should cultivate entrepreneurial and innovative talents by strengthening entrepreneurial psychological education of students. The scientific and reasonable entrepreneurial psychological education is helpful for college students to face the setbacks in the process of entrepreneurship, thereby helping them to achieve entrepreneurial success.

## Data Availability Statement

The raw data supporting the conclusions of this article will be made available by the authors, without undue reservation.

## Ethics Statement

The studies involving human participants were reviewed and approved by the Wenzhou Medical University Ethics Committee. The patients/participants provided their written informed consent to participate in this study.

## Author Contributions

All authors listed have made a substantial, direct and intellectual contribution to the work, and approved it for publication.

## Conflict of Interest

The authors declare that the research was conducted in the absence of any commercial or financial relationships that could be construed as a potential conflict of interest.
